# Supplementary material for: Knowledge of COVID-19 and preventive behaviors among waiters working in food and drinking establishments in Southwest Ethiopia
Source: PLoS One. 2021 Jan 25;16(1):e0245753. doi: 10.1371/journal.pone.0245753 (PMC7833477; doi:10.1371/journal.pone.0245753)
Supplement: S1 File — (DOCX) [file pone.0245753.s002.docx]

|  | **Part II: Risk Perception** |  | |  |
| --- | --- | --- | --- | --- |
|  | *How much do you agree or disagree with the following sta* | *tements?* | |  |
| 301 | Getting sick with the coronavirus can be serious | 1. Strongly disagree 2. Disagree 3. Neutral  3. Agree 5. Strongly agree | |  |
| 302 | My health will be severely damaged if I contract coronavirus | 1. Strongly disagree 2. Disagree 3. Neutral  4. Agree 5. Strongly agree | |  |
| 303 | It is not possible to recover from coronavirus disease | 1. Strongly disagree 2. Disagree 3. Neutral  4. Agree 5. Strongly agree | |  |
| 304 | Coronavirus causes deaths than other respiratory diseases | 1. Strongly disagree 2. Disagree 3. Neutral  4. Agree 5. Strongly agree | |  |
| 305 | If I caught with coronavirus, I cannot to manage daily activities | 1. Strongly disagree 2. Disagree 3. Neutral  4. Agree 5. Strongly agree | |  |
| 306 | People may stigmatize me if get sick due to coronavirus disease | 1. Strongly disagree 2. Disagree 3. Neutral  4. Agree 5. Strongly agree | |  |
| 313 | I think that you I will contract coronavirus if you do not take any preventive measure |  | 1. Strongly disagree 2. Disagree 3 Neutral  4. Agree 5. Strongly agree |  |
| 314 | I think that you I will contract coronavirus if you take preventive measure |  | 1. Strongly disagree 2. Disagree 3 Neutral  4. Agree 5. Strongly agree |  |
| 315 | I think that I will contract coronavirus if I come into contact with a coronavirus patient. | 1. Strongly disagree 2. Disagree 3 Neutral  4. Agree 5. Strongly agree | |  |
| 316 | I think that I might contract coronavirus even if I do not come into contact with a coronavirus patient. | 1. Strongly disagree 2. Disagree 3 Neutral  4. Agree 5. Strongly agree | |  |
| 317 | The coronavirus will NOT affect very many people in the area I'm currently living in | 1. Strongly disagree 2. Disagree 3 Neutral  4. Agree 5. Strongly agree | |  |
| 318 | My work exposes me more to coronavirus than another person |  | 1. Strongly disagree 2. Disagree 3 Neutral  4. Agree 5. Strongly agree |  |
|  | **(c) Perception of efficacy and self-efficacy** | |  |  |
| 324 | Do you think that you will manage to hand washing with water and sop or sanitizer frequently? | - - - 1. Certainly not 2. Probably not 3. Perhaps not – perhaps 4. Probably yes 5. Most certainly | |  |
| 325 | Do you think that you will manage to stay home? | Certainly not 2. Probably not 3. Perhaps not – perhaps 4. Probably yes 5. Most certainly | |  |
| 326 | Do you think that you will manage to maintain social distancing anywhere? | 1. Certainly not 2. Probably not 3. Perhaps not – perhaps 4. Probably yes 5. Most certainly | |  |
| 327 | Do you think that you will manage to use face mask always outside home /at work? | 1. Certainly not 2. Probably not 3. Perhaps not – perhaps 4. Probably yes 5. Most certainly | |  |
|  | **Barriers/hindering factors** |  | |  |
| 328 | What are the main barriers to implement (practice) key measures/ behaviors for COVID-19 prevention  (handwashing, social distancing and using facemask)? **(Mark all the barriers hindering factors)** | 1. No access to water, soap or hand sanitizer 2. No access/ Unbale to by mask 3. Difficult to maintain social distancing in context of my work 4. Takes too much effort (time, etc.) 5. I feel that too little information is provided about the measures 6. Other, namely…………………… | |  |

**Thank you for your participation!!!!**
